# Supplementary material for: Using logical constraints to validate statistical information about disease outbreaks in collaborative knowledge graphs: the case of COVID-19 epidemiology in Wikidata
Source: PeerJ Comput Sci. 2022 Sep 29;8:e1085. doi: 10.7717/peerj-cs.1085 (PMC9575845; doi:10.7717/peerj-cs.1085)
Supplement: Supplementary file 1 [file peerj-cs-08-1085-s001.odt]

Appendix A: SPARQL queries for the heuristics-based validation of epidemiological counts in Wikidata

| Task | SPARQL query |
| --- | --- |
| V1 | SELECT * WHERE {   ?x p:P31 [ps:P31 wd:Q3241045; pq:P642 wd:Q84263196].   ?x p:<PropertyID> [ps:<PropertyID> ?value; pq:P585 ?date].   FILTER(YEAR(?date) < 2019)   } |
| V2 | SELECT * WHERE {   ?x p:P31 [ps:P31 wd:Q3241045; pq:P642 wd:Q84263196].   ?x p:<PropertyID> [ps:<PropertyID> ?value; pq:P459 ?method].   FILTER NOT EXISTS {?method wdt:P279* wd:Q177719}   } |
| V3 | SELECT * WHERE {   ?x p:P31 [ps:P31 wd:Q3241045; pq:P642 wd:Q84263196].   ?x p:<PropertyID> [ps:<PropertyID> ?value; pq:P585 ?datep].   ?x p:<PropertyID> [ps:<PropertyID> ?value1; pq:P585 ?date].   FILTER(?value > ?value1)   FILTER(?datep - ?date = -1)   } |
| V4 | SELECT * WHERE {   ?x p:P31 [ps:P31 wd:Q3241045; pq:P642 wd:Q84263196].   ?x p:<PropertyID> [ps:<PropertyID> ?value; pq:P585 ?datep].   ?x p:<PropertyID> [ps:<PropertyID> ?value1; pq:P585 ?datef].   FILTER(?value = ?value1)   FILTER(?datep - ?datef = -2)   FILTER NOT EXISTS { ?x p:<PropertyID> [ps:<PropertyID> ?value2; pq:P585 ?date].                       FILTER(?date = ?datep + 1)                     } } |
| V5 | SELECT * WHERE {   ?x p:P31 [ps:P31 wd:Q3241045; pq:P642 wd:Q84263196].   ?x p:<PropertyID> [ps:<PropertyID> ?value; pq:P585 ?date].   FILTER(?value < 0)   } |
| V6 | SELECT * WHERE {   ?x p:P31 [ps:P31 wd:Q3241045; pq:P642 wd:Q84263196].   ?x p:P8049 [ps:P8049 ?h; pq:P585 ?date].   ?x p:P1603 [ps:P1603 ?c; pq:P585 ?date].   FILTER(?h > ?c)   } |
| V7 | SELECT * WHERE {   ?x p:P31 [ps:P31 wd:Q3241045; pq:P642 wd:Q84263196].   ?x p:P8011 [ps:P8011 ?t; pq:P585 ?date].   ?x p:P1603 [ps:P1603 ?c; pq:P585 ?date].   FILTER(?c >= ?t)   } |
| V8 | SELECT * WHERE {   ?x p:P31 [ps:P31 wd:Q3241045; pq:P642 wd:Q84263196].   ?x p:P1603 [ps:P1603 ?c; pq:P585 ?date].   ?x p:P1120 [ps:P1120 ?d; pq:P585 ?date].   FILTER(?c < ?d)   } |
| V9 | SELECT * WHERE {   ?x p:P31 [ps:P31 wd:Q3241045; pq:P642 wd:Q84263196].   ?x p:P1603 [ps:P1603 ?c; pq:P585 ?date].   ?x p:P8010 [ps:P8010 ?r; pq:P585 ?date].   FILTER(?c < ?r)   } |
| V10 | SELECT ?y ?date ((?count - ?c1) AS ?diff) WHERE { SELECT ?y ?c1 ?date (SUM(?c) AS ?count) WHERE {   ?x p:P31 [ps:P31 wd:Q3241045; pq:P642 wd:Q84263196].   ?x p:<PropertyID> [ps:<PropertyID> ?c; pq:P585 ?date].   ?x wdt:P361 ?y.   ?y p:<PropertyID> [ps:<PropertyID> ?c1; pq:P585 ?date].   } GROUP BY ?y ?c1 ?date } ORDER BY DESC(?diff) |

The SPARQL queries that were used for the Tasks defined in Table 2, to be run against the Wikidata Query Service available at [https://query.wikidata.org/](https://query.wikidata.org/) . Note that this query service has Wikidata-specific prefixes predefined, so they do not need to be re-stated in a query.
