# Supplementary material for: Using logical constraints to validate statistical information about disease outbreaks in collaborative knowledge graphs: the case of COVID-19 epidemiology in Wikidata
Source: PeerJ Comput Sci. 2022 Sep 29;8:e1085. doi: 10.7717/peerj-cs.1085 (PMC9575845; doi:10.7717/peerj-cs.1085)
Supplement: Supplementary file 2 [file peerj-cs-08-1085-s002.odt]

Appendix B: SPARQL queries for the validation of case fatality rate statements in Wikidata

| Task | SPARQL query |
| --- | --- |
| M1 | SELECT * WHERE {   ?x p:P31 [ps:P31 wd:Q3241045; pq:P642 wd:Q84263196].   ?x p:P3457 [ps:P3457 ?value; pq:P585 ?date].   FILTER((?value > 1) ││ (?value < 0))   } |
| M2 | SELECT ?x ?c ?d ?value ?date (ABS(?value - ?d / ?c) > 0.001 AS ?diff) WITH {   SELECT ?x {     ?x p:P31 [ps:P31 wd:Q3241045; pq:P642 wd:Q84263196].   } } as %outbreaks WITH {   SELECT ?x ?value ?date {     INCLUDE %outbreaks.     ?x p:P3457 [ps:P3457 ?value; pq:P585 ?date].   } } as %casefatalityrates WITH {   SELECT ?x ?d ?date {     INCLUDE %outbreaks.     ?x p:P1120 [ps:P1120 ?d; pq:P585 ?date].   } } as %deaths WITH {   SELECT ?x ?c ?date {     INCLUDE %outbreaks.     ?x p:P1603 [ps:P1603 ?c; pq:P585 ?date].   } } as %cases WHERE {   INCLUDE %casefatalityrates. INCLUDE %deaths. INCLUDE %cases. } ORDER BY DESC(?diff) |
| M3 | SELECT ?x ?c ?d ?date ((?d / ?c) AS ?m) WITH {   SELECT ?x {     ?x p:P31 [ps:P31 wd:Q3241045; pq:P642 wd:Q84263196].   } } as %outbreaks WITH {   SELECT ?x ?d ?date {     INCLUDE %outbreaks.     ?x p:P1120 [ps:P1120 ?d; pq:P585 ?date].   } } as %deaths WITH {   SELECT ?x ?c ?date {     INCLUDE %outbreaks.     ?x p:P1603 [ps:P1603 ?c; pq:P585 ?date].   } } as %cases WHERE {   INCLUDE %deaths. INCLUDE %cases.   FILTER NOT EXISTS {?x p:P3457 [ps:P3457 ?value; pq:P585 ?date].} } |

These SPARQL queries correspond to the Tasks M1, M2 and M3 that address heuristics concerning the case fatality rate m. The queries are specific to the Blazegraph SPARQL endpoint underlying the Wikidata Query Service, which provides some functions not available in standard SPARQL.
